# St. George's Hospital

**Published:** 1904-06-25

**Authors:** 


### ST. GEORGE'S HOSPITAL.

### SPECIAL MEETING TO CONSIDER THE QUESTION OF REMOVAL.

A Special Court of Governors of St. George's Hospital was held at the Westminster Palace Hotel on Tuesday last to receive the report of the committee appointed in March 1903 to consider the desirability of removing the hospital to a larger site.

The chair was occupied by the Right Hon. Lord Windsor, P.C., and there were also on the platform several members of the visiting staff and about 130 Governors.

The Secretary having read the notice convening the meeting, letters were read from the following Governors, who were unavoidably absent:—Lord Newlands, Lord Eustace Cecil, Mr. James Hozier, and Mr. F. C. Villiers, who expressed their objection to removal from the present site, and from Lord Egerton of Tatton, Sir William Bennett, and Mr. S. G. Lutwyche, who were equally strongly in favour of removal. Sir William Bennett wrote that since the public expression of his views on the matter nothing had occurred to alter his opinion that, provided the site could be advantageously disposed of, it would be better to remove, and adding that his views were fully in accord with those of the minority report of the committee. He had considered the matter from the broadest possible aspect, setting aside sentiment of personal convenience, and he trusted that a majority of the Governors would decline to allow money given with a view to meeting certain pressing needs to be diverted from their intention. Such a course would be wasteful, irrational, and useless. The following letter from Sir Henry Burdett had been received:—

"Owing to a serious accident which befell me in May, I have been kept a close prisoner in the country and unable to attend meetings or transact business until to-day. This has prevented me from attending the later meetings of the Committee appointed to consider the proposal to remove the hospital to a larger site, and accounts for the absence of my name from the report and final voting. Having taken the greatest interest in the whole of the proceedings of the Committee from the date of its appointment, and being in a position to know all the facts, as I find I shall not be well enough to attend the meeting of Governors on the 21st inst., I venture to send you the conclusions to which I have come on the questions which will then be brought to your notice.

"First of all it should be clearly understood that the Committee is in fact about equally divided, a conclusion which is borne out by the knowledge that Mr. West was in the chair, and that five members were unavoidably absent from the final meeting. I mention this point at the outset because it is well that the Governors should clearly understand that, despite the fact of the regrettable retirement of Sir William Bennett and Sir Isambard Owen, who were both in favour of removal to a new site, and the addition to the Committee of two members of the staff who were against removal, the result of the investigations has been to convince practically half the Committee that the course recommended in the minority report is in all the circumstances best for the future of St. George's Hospital.

"The position to-day is very simple, and may be stated in a few words. It is true that it will be possible, with the new site acquired, providing all the land now leased to the hospital is transferred to the Governors, to erect thereon a new hospital of about 300 beds, and to make it a modern hospital efficient in all respects. Such a result can, however, only be secured by clearing the site, if due regard is to be had to financial considerations, and the enforcement of the maximum economy in regard to both time and money. I take it that if this course were approved by the Governors the wisest step would be to close the hospital entirely for two years, at the end of which time the work could be resumed in the new buildings under the most favourable conditions. Meantime, temporary premises might be hired, where the work of the hospital could be carried on if such a step were thought desirable. In my judgment, it would be better to try and arrange with one of the large hospitals for facilities to be given to the students and for accommodation to be thus provided for the more urgent of the cases which under ordinary circumstances would be received in St. George's Hospital. The great objection to this course is clearly set forth on page 6 of the report, where it is shown by figures which cannot be questioned that if this step is taken it must involve a liability equal to the realisation of the whole £277,000 represented by the invested funds of the hospital, and, in addition, contributions from the public to the extent of from £70,000 to £100,000 more.

"There remains the alternative of disposing of the Governors' interests in the present site, the acquirement of a new site within the district, and the erection thereon of a modern hospital. If this latter course is taken, there can be no doubt that St. George's can be provided with a new hospital without further trenching upon its invested funds, and indeed that such a step should result in increasing those funds to the extent of something like £100,000. So far, then, as the financial aspects of the question are concerned, it is certain that men of business who have the true interests of the hospital at heart must vote, on the merits, in favour of removal.

"Dr. Arthur Latham, in his letter of April 28th last, has set forth what he considers to be the reasons in favour of retaining the hospital on its present site, despite the financial objections to this course. Dealing with the Medical School first, I must canvass Dr. Latham's conclusion, because I am fully convinced that in no circumstances can a sufficient portion of the present site be spared for the purposes of Medical School building to an extent sufficient to make that school as efficient or as important as it undoubtedly ought to be. If the hospital were removed to a site of at least 10 acres, St. George's would have an opportunity of possessing one of the best Medical Schools in London, and in view of the mistaken policy recently pursued by one of the great schools of Medicine in the Metropolis, it is, in my judgment, probable that in such conditions the Medical School of St. George's Hospital might become one of the greatest and most important in the Metropolis.

"Any scheme of removal must include the provision of accident wards in the neighbourhood of Hyde Park Corner, a fact which has been fully borne in mind by those responsible for the reports under your consideration. As to the payment of an honorarium to the medical staff, that is a practice which is growing up within the Metropolis, and cannot fail to have important economical results in the case of those hospitals which adopt it. The small annual payments made to the staff may for this reason be regarded as of little or no importance. It is not necessary for me to deal in detail with that portion of Dr. Latham's letter which is headed 'Financial,' in the course of which he anticipates the recurrence of national events with a frequency which is happily opposed to the teachings of history and the ordinary course of national affairs. I may, however, state, as the result of a careful investigation into the figures and facts relating to St. George's Hospital, extending over a period of 20 years, that I am convinced that the commanding site occupied by this hospital has had anything but a beneficial effect upon its finances, because the majority of people persuade themselves that a charity which can afford to stand upon so costly a site must necessarily be possessed of immense financial resources. This feeling is no doubt emphasised by the circumstance that the sites in its immediate vicinity are occupied by houses forming the residences of some of the most wealthy and prosperous of London's inhabitants.

"St. George's Hospital has recently spent, or is in course of spending, a sum which the late Treasurer puts at £200,000. I am aware that some people are never tired of asserting that the present buildings of St. George's Hospital are both healthy and convenient. This view cannot be supported by a personal inspection of the hospital as it is, in fact, to-day, for its wards contain many bad beds, the sanitary blocks are inadequate, and the wards and general buildings lack many essentials to efficiency, the absence of which must militate against the welfare of the patients and entail considerable additional cost upon the Governors each year.

"If due consideration is to be given to the business aspects and future welfare, reputation, and prosperity of St. George's Hospital, there seem to be but two alternatives, from which the Governors must make their choice:—(1) To appoint a committee of men of business to take steps to dispose of the present site at such a price as will enable the Governors to acquire a new site within the district, and to erect thereon a modern hospital, containing at least an equal number of beds to the present one; or (2) to raise £300,000 to enable the hospital to be rebuilt upon the site at Hyde Park Corner, the whole of which must then be acquired, and transferred in the first instance to trustees of the hospital.

"In my judgment, the adoption of the latter alternative must entail the closing of St. George's Hospital for at least two years, because to attempt to rebuild in sections would so increase the cost and prolong the period over which the work must extend that no wealthy person who considers the matter in all its bearings would be likely to contribute largely to a rebuilding fund unless it was made perfectly clear that the hospital would be closed during rebuilding.

The reports of the majority and the minority of the committee were then presented by Mr. A. W. West, who explained that, being in favour of the minority report, he only desired to draw attention to one point, viz., that no attempt had been made by the committee to dispose of the hospital's interest in the present site, or to negotiate for the present site, since the committee did not feel themselves in a position to do so. They thought the best course was to lay the reports before the Governors, and ask for a final decision.

Mr. C. G. Heathcote moved:—

> "That it is not desirable at the present time and under existing circumstances to remove St. George's Hospital from its present site."

Mr. Heathcote said no doubt some disappointment would be felt that the committee appointed at Grosvenor House in March 1903 had not been able exactly to fulfil the duty placed upon it. He thought it would be generally agreed that the time had come when it was absolutely necessary in the interests of the hospital that the doubt and uncertainty which for the last 18 months had hung over the future of St. George's should come to an end. (Hear, hear.) Such a state of things must be prejudicial to the efforts of those working in and for the hospital. The history of the question of removal was known to the Governors, and was contained in the reports and other papers circulated. It began practically rather more than a year and a half ago, and he believed the committee then constituted was superseded at the meeting held at Grosvenor House, when a committee vested with powers which he could not help thinking were too great was appointed to negotiate for the sale of the hospital and to deal with intending purchasers. He was thankful that the committee, shrinking from these powers, did nothing in that direction. A sub-committee of very able and intelligent business men, well versed in financial matters, was appointed to deal with removal on the one hand and rebuilding on the other, with general power to consider the question. In addition, the Medical School Committee were asked to report, the statement of their views being of the highest possible value. The tendency of the report of that special committee was undoubtedly against reconstruction on the present site and in favour of removal, while the Medical School Committee might be said, perhaps, to have been at one time in favour of reconstruction on the site, and absolutely opposed to removal. Although containing a great deal with which all would agree, the two reports were contradictory. No doubt many thought that if a sufficient sum of money to rebuild the hospital satisfactorily elsewhere were offered, the prospect of removal would be a tempting one, while if the finances were large enough to rebuild on the present site the proposal might be thought attractive

He would, however, dismiss the second alternative, because it was quite certain that the resources were not equal to reconstruction. The question, therefore, resolved itself into removal or non-removal. The removal of a hospital was a matter so serious that it could only be justified by very strong and cogent arguments. What, he asked, would be the materials naturally expected by the Governors from a committee appointed to consider the subject? In the first place, a definite, clear, and absolute statement of what there was for sale, clear proof that this was of great value, and that it could be sold at once for a good price. In the second place, a definite and clear offer from outside of some adequate sum to enable building to be carried out elsewhere. At any rate, it was reasonable to expect that grounds should be stated for the belief that a sum of money could be obtained for the site. The report, however, did none of these things. The speaker then proceeded to summarise the report in question, remarking that it tended to show that by removal in a south-western direction the convenience of a few patients might possibly be met, while it was frankly admitted that no effort had been made upon which action could be taken. Nor was the minority report more definite, both agreeing that there was no prospect of an adequate sum being obtained for what the hospital had to sell. He confessed to doubting the statement as to the sum said to be obtainable, as well as that referring to the estimated cost of rebuilding. Evidence, in his opinion, was lacking, and the statements were based upon hypothesis. That, he continued, was all the progress that had been made by what he might call the party of progress after a year's consideration. The reports contained, in fact, the opinions of the committee based upon assumptions. Were the interests of the hospital to be bartered away and the birthright sold on an hypothesis? Against these hesitating statements he set the report of those representing the Medical School, than whom no one was better qualified to come to a decision. They, at any rate, were unanimous in the opinion that it was inexpedient to move. (Hear, hear.) Proceeding, the speaker analysed the site, as set forth in the majority report, consisting of three portions held under different conditions, and added that no one had ventured to predict that an adequate sum could be obtained for the part under the control of the hospital alone. Dealing with the diagram illustrating the localities from which the in-patients were drawn, Mr. Heathcote said if locomotion were a serious difficulty in London he might form an argument in favour of removal to Chelsea or Fulham. In view, however, of existing facts, this need not be considered. The site they were asked to give up was, moreover, absolutely unequalled in any large town in the world for health, convenience, and access, and, not least important, for the position it occupied in the public eye. A large amount of support would be lost to the hospital if removed to the slums of Chelsea. Very large sums had been spent on the building in recent years, and it was of much greater value now than 20 years ago. No doubt it failed in some respects to come up to the ideal standard, but a hospital built to-day would be equally behind the times a few years hence. The necessary alterations could, he believed, be effected at a small cost. In conclusion, he appealed to the Governors not to abandon a site which must be the envy of almost every other hospital, and on which for 200 years the hospital had carried on its work with such excellent results.

Mr. C. T. Dent said what was wanted now was not arguments for or against removal, but a summing up of the whole question in order that a decision might be arrived at. The three cardinal points were: (1) to remove or to remain; (2) to come to an immediate decision, since nothing so sapped the vitality of a hospital as a state of indecision; and (3) to avoid any sort of compromise. He confessed to not seeing eye to eye with his medical colleagues in this matter. At the outset, he held that removal might be very advantageous, but, partly by the arguments of those in favour of retaining the present site, and still more by those of the opposite party, whose strength and authority he would be the last to underrate, he had been forced to see the wisdom of non-removal. As to the patients, the wards were filled very much more in virtue of a hospital's reputation than because of its position. (Hear, hear.) Of course, if it was possible to have a hospital in accordance with the views that happened to obtain at the particular moment when it was built, so much the better, but experience proved that in a few years the staff would not be satisfied with that. Although no one more than the staff would welcome the proposal to provide a new and up-to-date hospital, yet by a majority of more than ten to one the Medical School committee were in favour of remaining. The burden of proof rested on those desirous of moving, and they had no more solid reasons to show than conjecture—this was not too strong a term. He believed removal would be financially prejudicial, and very gravely disadvantageous to the efficiency of the hospital. The statement that the site was too valuable for a charity to remain on it was proof in itself of the value of that site for the purpose of the charity. (Hear, hear.) Finally, the money to enable the hospital to remain was obtainable provided the necessity for it could be shown. He was not speaking as a representative of the Medical School, but as a lay governor of many years' standing, and one to whom the hospital had been a kind of second home.

Mr. Robert Harrison defended the report of the Committee, and explained that it had been delayed owing to various reasons, one of which was that the Duke of Westminster, without whose sanction no decision with regard to a large portion of the site could be come to, only returned from South Africa early in the present year. Dealing with the report of the Medical School Committee, the speaker said two resolutions had been unanimously passed by that Committee—the first that it was undesirable to move, and that the Committee was of opinion that the time was close at hand when the hospital would require to be rebuilt on its present site; and the second, that no building should be commenced and placed on the land recently acquired in Knightsbridge unless the building were of such a kind that it would form a definite and convenient part of a new hospital, or until a complete plan of this new hospital had been carefully considered. The reasons for these decisions were set out in a memorandum, together with a synopsis of the arguments in favour of the present site. These were in the hands of the Governors. The members of the Medical Committee were naturally the persons best qualified to say what was wanted; but they made no suggestion as to how the necessary funds were to be raised to carry on the work. To purchase the site, pull down the present building and erect a new one, would require £120,000, and how was this sum to be obtained? Were the invested moneys of the hospital sufficient to recoup the expenditure? The income would be reduced by from £3,000 to £4,000 a year. Perhaps the Governors did not all realise that even now £10,000 a year beyond the income was being spent. The alternative course was to appeal to the public for £120,000, and it might possibly be forthcoming, although he thought the present not a good time to appeal, in view of the several large appeals before the public. Dealing with the sub-committee's report, the speaker said the site was one of the most valuable in London, and might be estimated to be worth from £12,000 to £15,000 a year. He considered that, as trustees of a charity supported by voluntary contributions, they were not justified, and it would be a grievous waste of money to rebuild on such a valuable site, and on this ground he would very much doubt the success of an appeal to the public. The estimated cost of a new hospital had been very carefully considered, having regard to the requirements set forth in the memorandum of the Medical School Committee, and the sum arrived at, £339,700, was based on the estimate of the architect, Mr. Percy Adams, and the figures might, he thought, be relied upon. The value of the present site, provided a purchaser could be found, was estimated at considerably more than the sum required to rebuild, allowing for a substantial balance in hand to add to the funds. At present rather more than 23 per cent. of the money subscribed was absorbed by the site, or 4s. 8d. out of every 20s. This would not be so if the hospital were removed to a less expensive site. Rates, moreover, swallowed up £1,039, a very much larger sum than was paid by other large London hospitals, and this would be increased by the acquisition of the property in Knightsbridge. He contended that while so vast a sum was "buried in the cellars" of the hospital the public should not be appealed to. From the patients' point of view—and the hospital existed for the poor—if over 50 per cent. came from Chelsea or Fulham, they would certainly gain by removal in that direction. At present the funds were being employed to a considerable extent to keep up the Medical School, and he did not see that removal would damage the school.

At this point there were cries of "Time," and the speaker concluded by saying that he hoped the Governors would decide that it would be wise, in view of the points raised, to move to a less expensive site—("No, no")—more accessible to the poor.

A Governor having remarked that he thought enough had been said,

Mr. Thorp said, that with regard to the suggestion to build on the small site in Knightsbridge, that would be tinkering, a method to which the MedicalS chool were strongly opposed. Temporary buildings had been suggested; he could not agree with this. Then, if the present site were retained, the Medical School said it would be necessary to rebuild in a few years. Where was the necessary money to come from? At least £340,000 was required, and it would cost more to rebuild the present hospital than to build another elsewhere. The hospital only possessed £270,000, and he failed to see how it would be possible to go to so great an expense. A point had been made of the cost of medical attendance; but £1,300 had been paid during 1903 to keep the school going, and he doubted whether the medical attendance would come to more. It was said that the school was falling off, and this was true, owing to the want of accommodation; he believed the number of students entering during the last three years had considerably decreased. On a new site a school could be built of any size required. With regard to the complaint that no definite offer for the site had been reported, there had been a tentative offer, but the committee had not felt themselves in a position to negotiate. He firmly believed that if the hospital were in a position to offer the site a sufficient sum could be obtained to enable a new hospital to be built; and it might not be generally known that a letter had been received asking that if the site were to be disposed of negotiations should proceed in conjunction with the Duke of Westminster. No proposal could be made, because it was not known how the hospital intended to act. ("Time, time.")

The Chairman asked whether it was the wish of the meeting to take a vote, and received an affirmative reply. The resolution was then put, and carried by a large majority with applause and acclamation.

The following resolution, proposed by Mr. Heathcote and seconded by Mr. Dent, was considered as a corollary to the first, requiring no comment:—

"That the present is not a favourable opportunity for an appeal to the public for funds to rebuild the hospital on the present site."

Sir Isambard Owen rose to protest. He did not doubt the literal truth of the proposition, but thought it would be very injudicious to pass a resolution to that effect. The Governors had decided that they would not have the hospital removed, and he would say emphatically that they must at once face the question of rebuilding it at Hyde Park Corner. It would do no good to pass such a resolution, since the public must be appealed to at the very first opportunity. The state of the hospital was urgent. Speaking in the name of his colleagues, he urged the necessity for certain extensions, if the work was to be carried on satisfactorily, and these could not be properly made until the whole question of rebuilding had been adequately considered. The question must be taken vigorously in hand by the Governors at once, and he strongly deprecated the passing of any resolution which would tie their hands.

The resolution was then put, and lost by a large majority.

The next resolution on the agenda was: "That the newly-acquired site in Knightsbridge should be utilised for the immediate requirements of the hospital as soon as occasion shall serve."

Some little discussion on the platform resulted in the announcement that the resolution would be withdrawn, but eventually it was proposed by Mr. Heathcote, in whose name it stood. Seeing that there was an amendment to follow, Mr. Heathcote supposed it was desirable that the meeting should be asked to express an opinion, although he did not know that it was essential to decide at once what use should be made of the newly-acquired site. It would, however, strengthen the hands of the governing body to know the opinion of the Governors, since £60,000 had been given for the site, and it must be utilised. The property was at present occupied by tenants whose leases would eventually fall in or otherwise terminate, and the sanction of the Governors was asked in order that when the property was available it might be put to the best purpose possible.

Dr. Latham proposed to add after the word "serve" the words "provided that no building be commenced and placed on the land recently acquired in Knightsbridge unless the building is of such a kind that it will form a definite and convenient part of a new hospital, or until a complete plan of this new hospital has been carefully considered." He believed the amendment would be accepted by the mover and seconder; it was proposed with the object of preventing tinkering and waste of money, and to ensure that whatever was built on that portion of the property should eventually form part of the new hospital.

The Chairman asked if there were any opposition to the amendment, and Dr. Dickinson having remarked that he would rather leave the Governors with a free hand, unfettered with regard to the future, Mr. Timothy Holmes agreed. It seemed, he said, to have been assumed that the whole structure should be pulled down and built up again from the ground. The question of rebuilding had never been formally considered by the Medical Committee, and it struck him as a very admirable way of wasting money. About £2,000 had been spent on the present structure since he joined as a student, and all that money would be absolutely wasted if it should seem necessary to pull the present hospital down and build another. The necessity had never been shown, and he thought it should be very carefully considered by the Medical Committee before any proposal of the sort was made. The new site could not be dealt with for a good many years—the longest tenancy was from 12 to

14 years (30 years, he was reminded by a speaker); at any rate, the lease had some years to run, and the site could not be dealt with just yet. He counselled the Governors not to be in a hurry, or to fetter themselves with resolutions. He was quite in favour of the resolution as it stood.

Dr. Isambard Owen said he had been very carefully into the plans of the present building, and into the question of the numerous inconveniences, and he was quite confident that no really good building could be made out of it by tinkering it and adding fresh portions. All this had been said by the late Mr. Charles Hawkins and others twelve years ago, and if their advice had been taken then there would have been none of the waste of money now going on in expensive alterations, which did not make it a really first-class hospital.

The Chairman put the resolution as amended, and it was carried.

Mr. Heathcote moved:—

"That it is desirable at once to negotiate with the Duke of Westminster for the purchase of the leaseholds on the terms proposed in the letters from his Grace's solicitors of March 10th, 1899, of January 6th, 1904, at the price of £23,700."

The Governors having decided that the hospital should remain where it was, Mr. Heathcote said it was absolutely essential that they should acquire what were practically the freeholds of that portion facing Grosvenor Place of which the lease expired in 1906.

Mr. Dent having seconded, the resolution was carried unanimously.

A letter received by Dr. Latham from Dr. Robert Barnes was then read, cordially approving of the resolution based upon the report of the Medical School, and stating that he was prepared to subscribe £1,000 for the promotion of the school improvements.

This was received with applause.

A list of new Governors having been read by the Secretary, the proceedings terminated with a vote of thanks to the Chairman.

